# A rare case of perivascular epithelioid cell tumor (PEComa) of the greater omentum

**DOI:** 10.1186/s12957-018-1407-5

**Published:** 2018-06-19

**Authors:** Koichi Okamoto, Yuka Okada, Kohei Ohno, Takahiro Yagi, Mitsuo Tsukamoto, Takuya Akahane, Ryu Shimada, Tamuro Hayama, Takeshi Tsuchiya, Keijiro Nozawa, Keiji Matsuda, Tsuyoshi Ishida, Fukuo Kondo, Yojiro Hashiguchi

**Affiliations:** 1 Department of Surgery, Teikyo University School of Medicine, 2-11-1 Kaga Itabashi-ku, Tokyo, 173-8605 Japan; 2 Department of Pathology, Teikyo University School of Medicine, Tokyo, Japan

**Keywords:** Perivascular epithelioid cell tumor, PEComa, Human melanin black 45, HMB45, Omental primary tumor, Angiomyolipoma, Omental PEComa

## Abstract

**Background:**

A tumor composed exclusively or predominantly of human melanin black 45 (HMB45)-positive epithelioid cells is called a perivascular epithelioid cell tumor (PEComa). We report a very rare case of a PEComa of the greater omentum.

**Case presentation:**

MRI conducted to examine the orthopedic disease of the patients, a 49-year-old Japanese woman, also identified a tumor in her pelvis. A CT scan revealed a tumor mass on the right side of the pelvic floor and clear nutrient vessels originating from the splenic and celiac arteries. An omental primary tumor or accessory spleen was thus suspected, and tumor resection was performed. The tumor was a light brown solid tumor with a smooth margin, measuring 5.2 × 3.8 × 3.5 cm. Histopathologically, the tumor was composed mainly of spindle and epithelioid cells, and large and small blood vessel formation was observed. In the immunohistochemical staining, tumor cells were positive for human melanin black 45 (HMB-45) and Melan-A and partially positive for alpha-smooth muscle actin. The final diagnosis was PEComa of the greater omentum.

**Conclusions:**

Although omental PEComa is very rare, it should be considered as a differential disease of an omental primary tumor.

## Background

A perivascular epithelioid cell tumor (PEComa) is a rare mesenchymal tumor composed of epithelioid cells characterized by histological and immunohistochemical evidence of both smooth muscle and melanocytic differentiation [1]. In immunohistochemical staining, the melanocyte marker HMB-45 (human melanin black 45) is the most sensitive (92% positive); Melan-A is the next most sensitive. The smooth muscle marker smooth muscle actin (SMA) is found in 80% of PEComas [2]. PEComas have been reported to occur at various sites such as gynecologic sites, ureter, intestinal tract, bone, and skin [2–6]. However, there are few reports of primary omental PEComa.

Although primary tumors of the greater omentum are rare, there are several reports of malignant tumors of this type [7]. Therefore, in the treatment of an omental primary tumor, the preoperative differential diagnosis is important, and it is necessary to consider PEComa as one of the differential diagnoses. Here, we describe a rare case diagnosed as an omental PEComa postoperatively.

## Case presentation

**Fig. 1:**
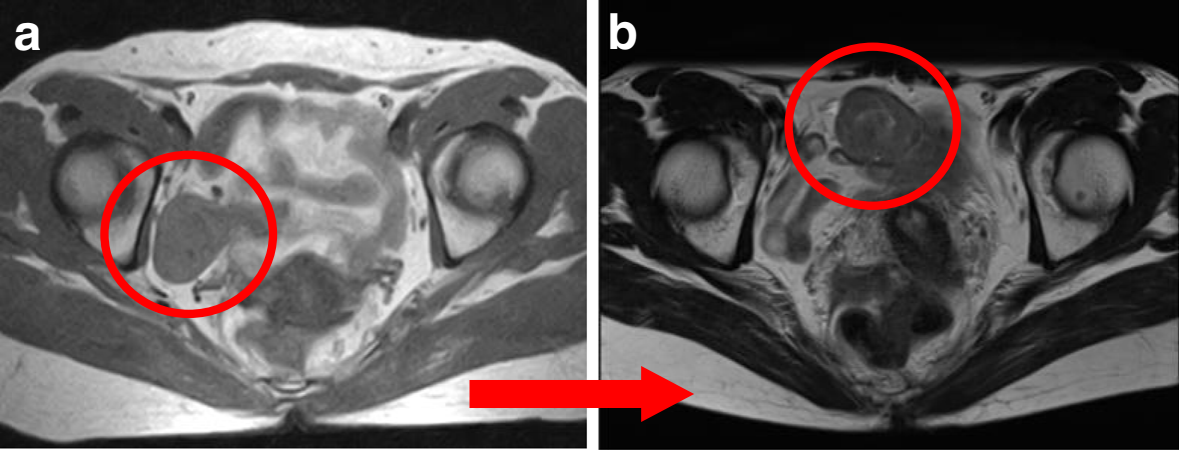
Preoperative pelvic T1-weighted MRI findings. A tumor mass of 37 mm in size was found in the pelvis, and a mobile lesion in the peritoneal cavity was observed by comparing the image taken at the referral hospital (**a**) with the image taken at our hospital (**b**). The tumor appeared at a mildly higher signal than the muscle

A 49-year-old Japanese woman underwent an MRI examination in the referring hospital for an assessment of the orthopedic disease of her right hip joint, and the MRI revealed a tumor in her pelvis. She was referred to our hospital, where an MRI examination again showed tumor mobility (Fig. 1), and a tumor derived from the intestinal tract was suspected.

**Fig. 2:**
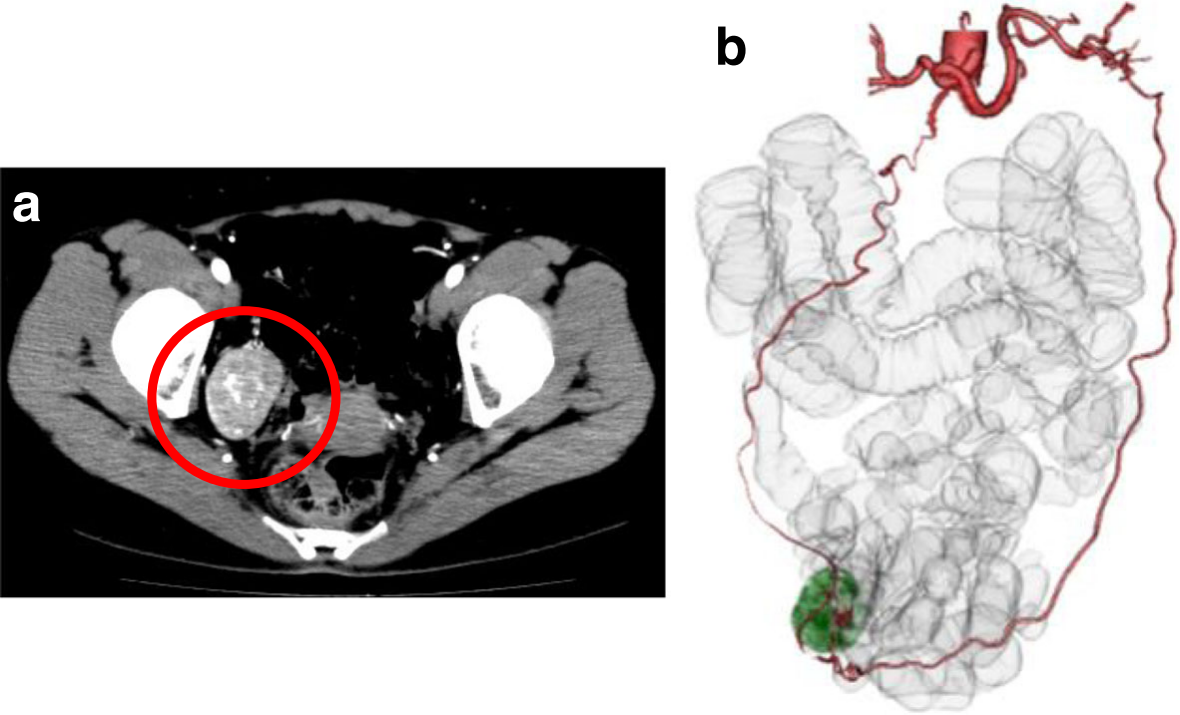
Contrast-enhanced CT image findings. **a** A 45-mm-large tumor mass on the right side of the pelvic floor. Inhomogeneous mineralization which was somewhat borderless was recognized inside. **b** Clear nutrient vessels originating from the splenic artery were considered omentum branches

**Fig. 3:**
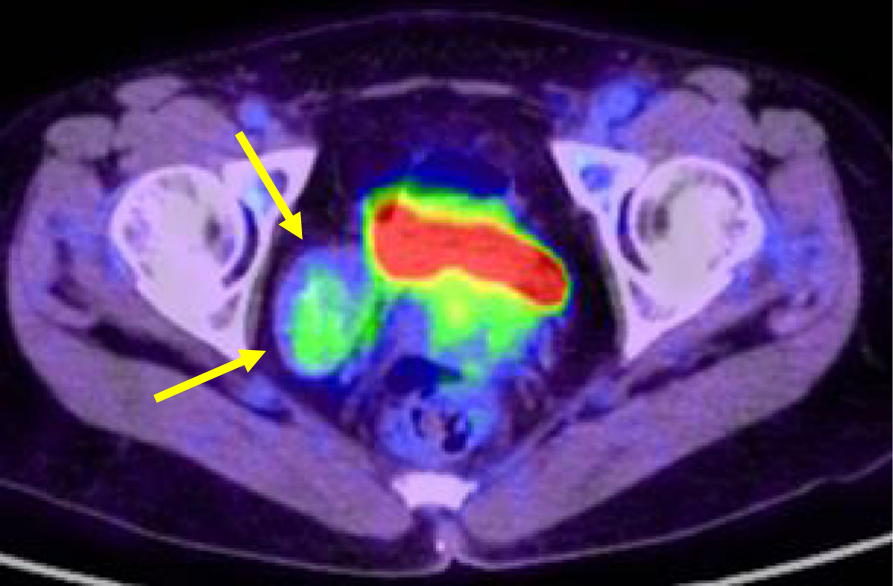
^18^F-fluoro-2-deoxyglucose positron emission tomography/computed tomography (^18^F-FDG-PET/CT) finding. The SUV max of the tumor was 2.56 in the early phase and 2.49 in the late phase, and no abnormal accumulation in the tumor (yellow arrow) was observed

In the contrast CT image (Fig. 2), a mass approx. 45 mm in size was found on the right side of the pelvic floor, and in the blood vessel construction image, the tumor was nourished by vessels from the splenic artery to the greater omentum. In ^18^F-fluoro-2-deoxyglucose positron emission tomography/computed tomography (^18^F-FDG-PET/CT), no abnormal accumulation was observed (Fig. 3). The results of complete blood cell counts and biochemical tests were all normal. Cancer antigen 19-9 (CA19-9) showed a mild elevation at 37.8 U/mL (normal range < 37.0 U/mL), and carcinogenic embryonic antigen (CEA) was normal at 0.8 ng/mL (normal range < 5.0 ng/mL). Based on all of these results, the preoperative diagnosis was a suspected omental primary tumor or accessory spleen.

**Fig. 4:**
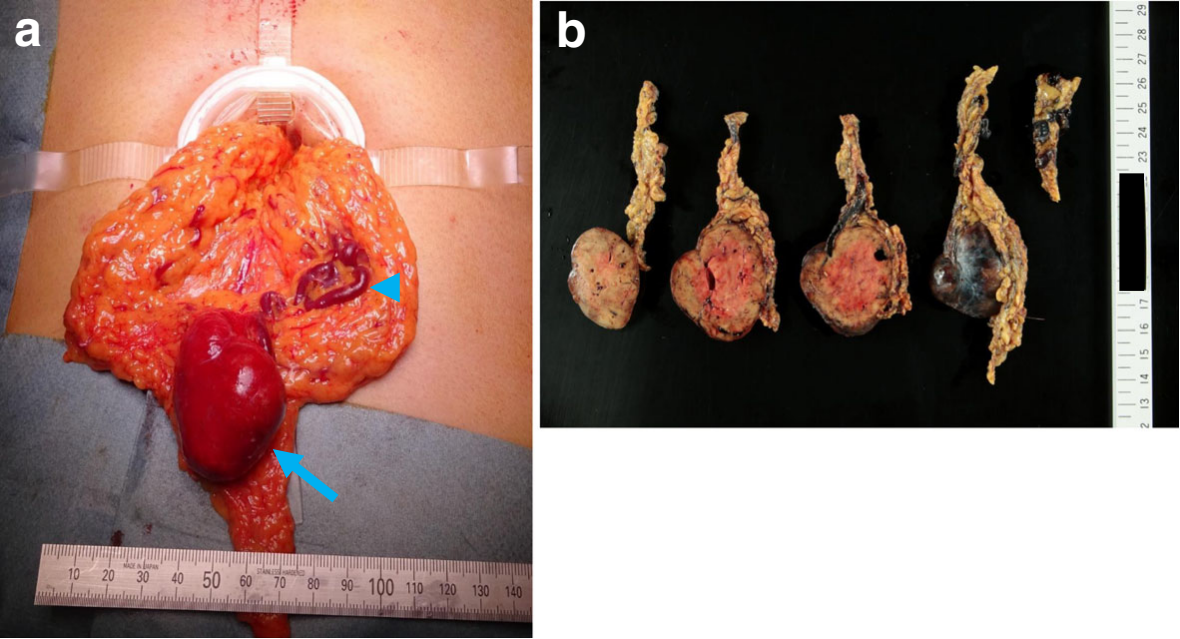
**a** Intraoperative finding. Greater omentum and omental tumor induced outside the body. About 5 cm large omental tumor (arrow) and nutrient blood vessel (arrowhead) inside the greater omentum. **b** Resected specimen. A light brown solid tumor with a clear and smooth border with a size of 5.2 × 3.8 × 3.5 cm

There was a danger of torsion irrespective of the presence or absence of malignancy, and the patient desires surgery to remove the tumor; the surgery was conducted concurrently with the diagnosis and treatment. The surgery was a single-incision laparoscopic-assisted greater omental tumor resection. A longitudinal incision was made approx. 4 cm around the umbilicus. A multichannel port (x-Gate®, Sumitomo Bakelite, Tokyo) was inserted in the wound. Observation of the abdominal cavity revealed a red and solid tumor mass of approx. 4 cm. Adhesion with the surrounding tissue was not observed. The greater omentum and the tumor were guided out of the body through the gate hole (Fig. 4a), and the tumor was excised together with the greater omentum. The tumor, a light brown tinged solid mass with a smooth border, was 5.2 × 3.8 × 3.5 cm in size with a well-defined border (Fig. 4b).

**Fig. 5:**
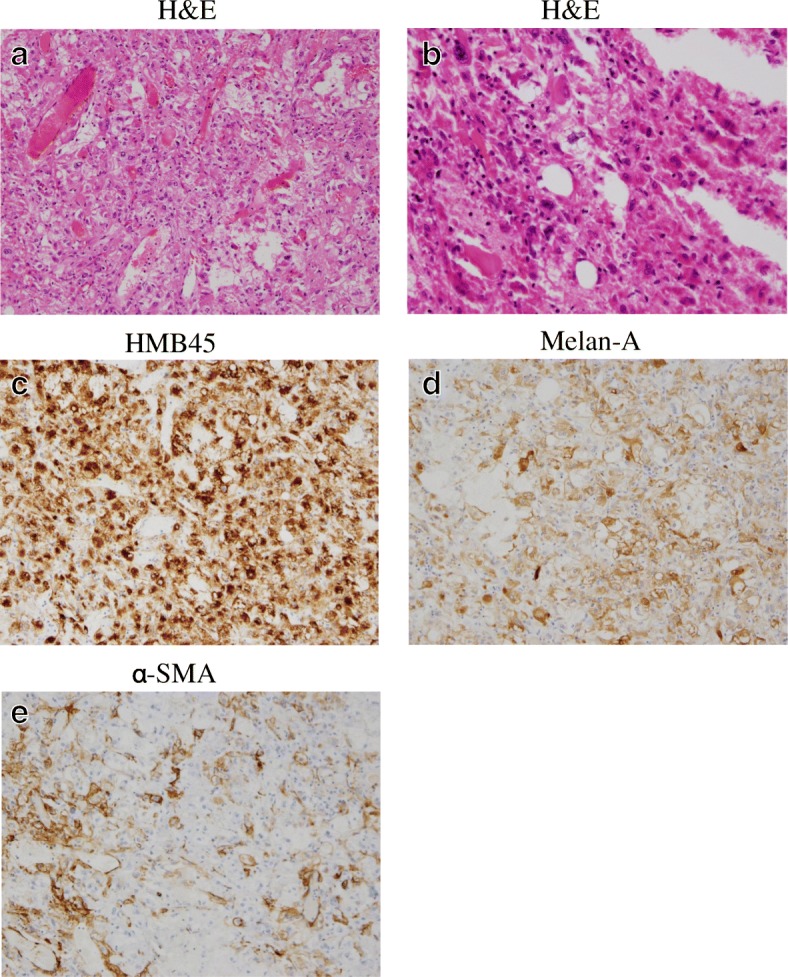
Histopathological findings. **a**, **b** Hematoxylin and eosin (H&E) staining photomicrographs. Immunohistochemical staining photomicrographs for **c** HMB45, **d** Melan-A, and **e** α-SMA. Photomicrographs were taken under a × 20 objective and a × 10 ocular lens (**a**, **c**, **d**, **e**) and under a × 40 objective and a × 10 ocular lens (**b**)

Histopathologically, the tumor involved large and small blood vessels (Fig. 5a), and tumor cells with eosinophilic cytoplasm were increased in number, in sheet form (Fig. 5b). In immunohistochemical staining, the tumor cells were found to be positive for HMB45 (Fig. 5c), Melan-A (Fig. 5d), and α-SMA (Fig. 5e), but negative for CD34, Desmin, c-kit, and s-100.

Based on the above results, the tumor was diagnosed as an omental PEComa. There has been no recurrence at 16 months after the surgery.

## Discussion

The image findings of primary PEComa have been reported to be nonspecific [8]. In this patient’s case, we considered omental liposarcoma, PEComa, teratoma, and accessory spleen as the preoperative diagnosis, considering the MRI findings and partial calcification in CT. Omental tumors have been reported to cause torsion [9–11]. Indeed, in our patient’s case, the possibility of twisting was considered because the preoperative diagnosis of omental PEComa was difficult and because the approx. 5-cm tumor was located at the tip of the greater omentum, which also presents a risk of torsion of the omentum. The surgery was thus carried out concurrently with the patient’s diagnosis and treatment.

Omental PEComas are very rare. To the best of our knowledge, only two prior cases of omental PEComa or perivascular epithelioid cell tumor have been reported. Our literature search using PubMed with “omental PEComa” or “omental perivascular epithelioid cell tumor” as keywords revealed that only one case of omental PEComa was reported between 1950 and 2018; the case was an omental angiomyolipoma reported by Takamura et al. [12]. That case was quite different from our patient’s because multiple angiomyolipomas coexisted in the liver. In addition, the case previously received right nephrectomy and enucleation of the left kidney for angiomyolipomas of both kidneys 11 years ago. Another case could not be retrieved by PubMed search with the above keywords, but the case was included in the 26 perivascular epithelioid cell neoplasm cases reported by Folpe et al. [2]. However, details are unknown. Our case is the first single PEComa of the greater omentum.

Laparoscopic surgery is less invasive compared with open surgery [13–16]. In addition, umbilical single-incision laparoscopic surgery (SILS) is cosmetically superior to open surgery [17]. As a malignant PEComa that caused intraperitoneal seeding has been reported in the past, it is necessary to firmly search the abdominal cavity during surgery [18]. For these reasons, in a mobile omental tumor, it is a good indication of SILS. In our case, SILS was chosen because the patient emphasized the cosmetic result of the wound. This is the first report of omental PEComa excised by SILS which is cosmetic and enables simultaneous observation in the abdominal cavity and tumor resection.

Since PEComa is a rare disease, there are few reports on criteria of malignancy, but in 2005, Folpe et al. proposed a classification that divides PEComa from benign to malignant into three categories: benign, uncertain malignant potential, and malignant (Table 1) [2]. According to the classification, our patient’s case applies only to a factor of “size > 5 cm,” so it can be considered as “uncertain malignant potential.” Our patient has been recurrence-free for 16 months since the surgery, as confirmed by CT follow-up.

**Table 1:** Classification of PEComas proposed by Folpe et al

	Benign	Uncertain malignant potential	Malignant
Criteria	No worrisome features	Nuclear pleomorphism/multinucleated giant cells only	Two or more worrisome features
	Size < 5 cm	or	Size > 5 cm
	Non-infiltrative	Size > 5 cm only	Infiltrative
	Non-high nuclear grade and cellularity		High nuclear grade and cellularity
	Mitotic rate ≦ 1/50HPF		Mitotic rate ≧ 1/50HPF
	No necrosis		Necrosis
	No vascular invasion		Vascular invasion

## Conclusions

We presented the case of a primary greater omental PEComa with uncertain malignant potential in a 49-year-old woman. Although omentum PEComas are very rare, omental tumors following a malignant course must be treated correctly and should be considered a differential diagnosis.
